# Acquired mutations in transcriptional regulator *hns* and lipopolysaccharide biosynthesis and modification genes impact colistin resistance and fitness of *mcr*-carrying *E. coli*

**DOI:** 10.1128/aac.00457-26

**Published:** 2026-06-18

**Authors:** Anna Schumann, Ian P. O’Keefe, Robert K. Ernst, Martin Wiedmann

**Affiliations:** 1 Department of Food Science, Cornell University 5922, Ithaca, New York, USA; 2 Graduate Field of Biomedical and Biological Sciences, Cornell University 5922, Ithaca, New York, USA; 3 Department of Microbial Pathogenesis, School of Dentistry, University of Maryland, Baltimore, Maryland, USA; 4 Departments of Biochemistry and Molecular Biology, School of Medicine, University of Maryland 271624, Baltimore, Maryland, USA; Universita degli Studi di Roma La Sapienza, Rome, Italy

**Keywords:** H-NS, LpxC, *Escherichia coli*, colistin, evolution, mcr

## Abstract

Gram-negative bacteria can develop resistance to the last-resort antimicrobial colistin through the acquisition of *mobile colistin resistance* (*mcr*) genes or chromosomal mutations. Bacteria carrying *mcr* variants do not always have colistin minimum inhibitory concentrations (MICs) above the clinical breakpoint, and there is a gap in understanding how *mcr*-carrying bacteria adapt to colistin selective pressure. To address this gap, we propagated *Escherichia coli* strains carrying *mcr-3* or *mcr-9*, which have been reported to confer different levels of colistin resistance and fitness costs, and a control strain lacking an *mcr* gene in the presence of increasing concentrations of colistin. While the *mcr-9* and control strains maintained their baseline colistin MICs, the colistin MIC of the strain expressing *mcr-3* increased, alongside acquisition of nonsynonymous mutations in genes involved in lipopolysaccharide biosynthesis and modification. Analysis of individual *mcr-3* strains that evolved during the experiment revealed that mutants exhibited improved competitive performance under colistin selection, but showed reduced fitness in standard media, or in the presence of human serum. Nonsynonymous *lpxC* and *hns* mutations were significant predictors of overall fitness and bacterial growth, but were infrequent among wild-type *E. coli* isolates, and even less frequent in *mcr-*positive wild-type *E. coli*. Our results suggest that *mcr*-expressing *E. coli* can evolve to show increased colistin MICs; however, fitness costs may prevent these mutations from being established in natural populations. Moreover, *E. coli* might control *mcr-3* expression through the nucleoid protein H-NS, providing novel insights into bacterial regulation of *mcr* expression.

## INTRODUCTION

Colistin is an important last-resort antimicrobial for multidrug-resistant (MDR) gram-negative pathogens (1). Following the discovery of the first *mobile colistin resistance* (*mcr*) gene (2), many countries discontinued the use of colistin as a growth promoter in animals and reserved colistin for use in human medicine (3). Despite these measures, colistin resistance has grown more prevalent with increased use in human medicine (4). Understanding colistin’s mechanism of action is therefore essential to determine resistance development. Mechanistically, antimicrobial activity of colistin is mediated by electrostatic attraction between its positively charged head group and the negatively charged phosphate groups of the lipopolysaccharide (LPS) component lipid A (5, 6). Two mechanistically distinct forms of acquired resistance have been reported. First, bacteria can acquire mutations in chromosomally encoded two-component regulatory systems, including *phoPQ* and *pmrAB*. PmrAB regulates the expression of several lipid A-modification enzymes like EptA or ArnT, which add phosphoethanolamine (pEtN) or L-aminoarabinose, respectively, to the terminal phosphate moieties of lipid A; this reduces the negative charge on lipid A, thereby weakening colistin’s binding and leading to resistance (1, 7). Secondly, bacteria can acquire *mcr* genes, which confer colistin resistance through a similar enzymatic mechanism as chromosomally encoded EptA (4, 5).

A number of studies have further examined the acquisition of colistin resistance in clinical strains. For example, a longitudinal study (8) that monitored *Acinetobacter baumannii* infections before and during colistin treatment showed that *A. baumannii* strains acquired chromosomal mutations that led to evolved colistin resistance. Chromosomal mutations are of concern during individual treatments; however, these mutations are less of a concern from a global public health perspective as they can only be transmitted vertically but not horizontally. Conversely, *mcr* genes are primarily located on plasmids, are easily transmitted horizontally, and also decrease susceptibility to colistin (4). To date, 10 different families of *mcr* variants have been discovered, all of which decrease susceptibility to colistin at different levels (9). Interestingly, *mcr-*carrying bacteria often exhibit colistin MICs around 2 μg/mL, which falls below the clinical breakpoint set by the Clinical Laboratory and Standards Institute (CLSI) as ≥4 µg/mL (9, 10); however, no “standard” cutoff value exists for colistin resistance, and appropriate cut-off values have been debated widely (10). While *mcr*-carrying bacteria may sometimes not be considered colistin resistant, previous studies showed that bacteria carrying *mcr-1* can acquire additional mutations through gradual exposure to increasing colistin concentrations that result in increased colistin MICs (11, 12).

While several studies (11, 12) have assessed how *mcr-1*-carrying strains have evolved under colistin selective pressure, no study has assessed how other *mcr* variants evolve under similar experimental conditions. Assessing how bacteria carrying *mcr* variants other than *mcr-1* is important because previous studies have demonstrated that different MCR variants have distinct phenotypes; for example, a study (13) identified phenotypical and biochemical differences between MCR-1, -3, and -9, including but not limited to differences in levels of conferred colistin resistance, cell viability defects, and preference as to which phosphate of lipid A is modified by a given MCR enzyme. We hypothesized that bacteria carrying *mcr-3* and *mcr-9* will develop increased colistin MICs but will show distinct changes in genotypes and phenotypes after exposure to colistin. To test our hypothesis, we used an *in vitro* evolutionary experimental approach to study the acquisition of mutations in *Escherichia coli* strains carrying single copies of *mcr-3* or *mcr-9* under their native promoters, in response to colistin selective pressure. We also assessed whether acquired mutations persisted after the selective pressure was removed. Our focus was *mcr-3* and *mcr-9*, which are commonly found in wild-type (WT) bacteria (13, 14) and which modify opposite lipid A phosphate groups: MCR-3 targets the 4′-phosphate, while MCR-9 modifies the 1-phosphate. These two *mcr* variants confer differing colistin resistance levels—high for MCR-3 and low for MCR-9—and have previously been reported to exhibit contrasting effects on cell viability, with MCR-9 reducing and MCR-3 not impacting cell viability (13).

## RESULTS

### *E. coli* MG1655 carrying *mcr-3* and *mcr-9* under control of their native promoters show distinct colistin resistance profiles

**Fig 1:**
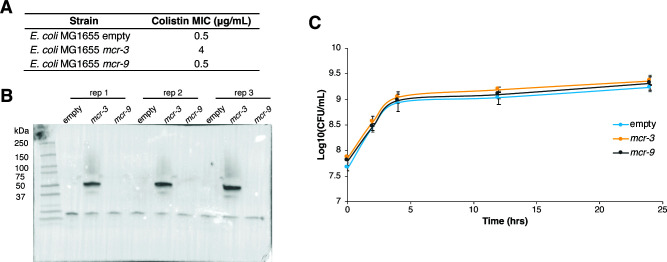
Colistin MIC, protein expression, and bacterial growth data of parent strains. (**A**) Colistin MICs of *E. coli* MG1655 strains carrying the empty *Tn7* transposon (abbreviated as “empty” in panels B and C), or transposons with *mcr-3* or *mcr-9* genes and their respective native promoters; designated *E. coli* MG1655 *mcr-3* and *E. coli* MG1655 *mcr-9*, respectively (for all three strains, all three biological replicates had the same MICs). (**B**) Western blot analysis of MCR protein expression from their MYC tag. (**C**) Bacterial growth of all three parent strains grown in brain heart infusion (BHI) broth was assessed over 24 h; data represent means of three biological replicates, and error bars represent the standard error of the mean.

To investigate the evolution of *mcr-*carrying strains under colistin selective pressure, we generated *E. coli* strains with single copies of *myc*-tagged *mcr-3* or *mcr-9* under their respective native promoters. These strains served as the starting points for our *in vitro* evolutionary experiments and will be referred to as “parent strains.” We used *E. coli* MG1655 as a base strain due to its ease of genetic manipulation, minimal laboratory-adaptive mutations (15), and fully annotated, publicly available genome. Colistin MICs and bacterial growth of the *mcr* parent strains were assessed in comparison to an empty control strain (i.e., MG1655 carrying the Tn7 transposon without an *mcr* gene) to understand the baseline phenotypes of the parent strains (Fig. 1). The *E. coli* MG1655 *mcr-3* parent strain showed a colistin MIC of 4 μg/mL (Fig. 1A), whereas the *mcr-9* parent strain showed the same colistin MIC as the empty control (0.5 μg/mL). These results suggest that *mcr-3* increases the colistin MIC eightfold, while *mcr-9* does not change the colistin MIC of *E. coli* MG1655 (Fig. 1A).

While Western blot analysis confirmed MCR-3 expression, no MCR-9 protein expression was observed (Fig. 1B). However, all three parent strains exhibited identical growth patterns (Fig. 1C). The absence of MCR-9 expression may be due to an inverted repeat sequence within its native promoter region, as previously described (15). Because natural *mcr-9*-carrying isolates often appear to be colistin susceptible when tested (16), we chose to proceed with the *in vitro* evolutionary experiment despite not having detected *mcr-9* expression in our strain (Fig. 1B). We hypothesized that exposure to subinhibitory levels of colistin could either induce *mcr-9* expression or generate mutants that express *mcr-9*, as previous studies have shown that *mcr-9*-carrying *E. coli* only showed pEtN-modified lipid A in the presence of colistin (17).

### *E. coli* MG1655 populations expressing *mcr-3* develop increased colistin MICs when exposed to colistin

**Fig 2:**
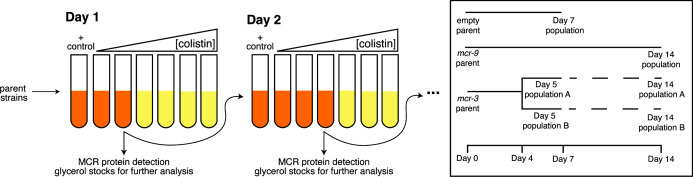
Set up of the *in vitro* evolutionary experiment. Propagation for the *in vitro* evolutionary experiment involved inoculating BHI broth with multiple different colistin concentrations and a non-colistin-containing growth control. Cultures were passaged after 24 h. Each subsequent passage, fresh cultures were inoculated from the population with the highest colistin concentration that showed growth. Each culture used for passaging was saved for downstream analyses. The box on the right shows the propagation timelines for each of the three parent strains. Solid lines represent propagations in the presence of colistin, dashed lines represent propagations in the absence of colistin.

To determine how *E. coli* MG1655 strains carrying *mcr-3* or -*9* adapt when exposed to increasing concentrations of colistin, we exposed both parent strains, as well as our empty control strain, to increasing concentrations of colistin in brain heart infusion (BHI) broth. We chose to use BHI rather than Mueller-Hinton II (MHII) broth (the media used in MIC assays) because we consider it to be more representative of *in vivo* conditions than MHII, as it is a complex medium using calf brain and beef heart infusion in its formulation. During each passage of our *in vitro* evolutionary experiment, every strain was exposed to several different concentrations of colistin; we also included a growth control without colistin in each passage (Fig. 2). Each subsequent passage was started from the previous culture with the highest colistin concentration that showed growth, which was subsequently frozen for assessment of population-based phenotypes (Fig. 2). The empty and *mcr-9* parent strains were propagated in the presence of colistin for 7 and 14 days, respectively. The *mcr-*3 strain was propagated in the presence of colistin for 7 days but split into two separate propagations after day 4 because the day 4 culture grew at a colistin concentration 8× higher than the parent strain’s colistin MIC. After 7 days in the presence of colistin, each *mcr-3* culture was further propagated without colistin for another 7 days to determine how phenotypes and genotypes evolved in the absence of colistin selective pressure. Since each sample from the *in vitro* evolutionary experiments may contain a mixture of mutants derived from the parent strains, we will refer to them as “populations” moving forward.

**Fig 3:**
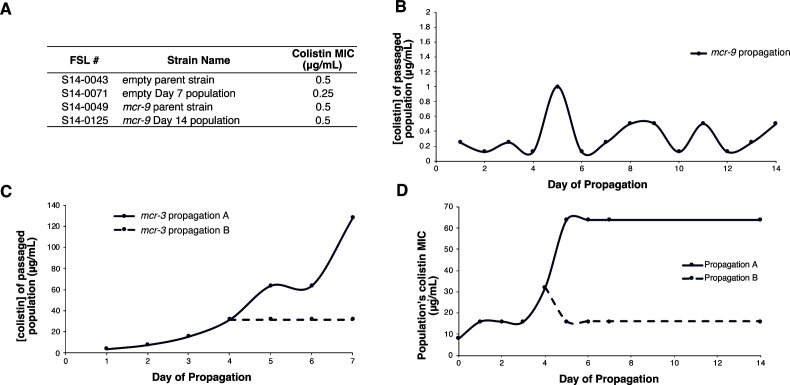
Changes in colistin MICs of *E. coli* MG1655 *mcr-3* and *mcr-9* populations. (**A**) Median colistin MICs of the parent strain and the population after the last day of propagation during the *in vitro* evolutionary experiment are shown for *E. coli* MG1655 empty and *mcr-9* populations (*n* = 3). (**B, C**) Colistin concentrations of each passaged population for *E. coli* MG1655 *mcr-9* (**B**) and *mcr-3* (**C**) populations during the *in vitro* evolutionary experiment in the presence of colistin. These values represent the highest colistin concentrations at which growth was detected for each individual passage. Colistin concentrations of each passaged *E. coli* MG1655 empty control population can be found in Fig. S1A. (**D**) Colistin MICs for each collected *E. coli* MG1655 *mcr-3* population from the propagations shown in panel B were tested in MIC assays according to CLSI standards in MHII broth (*n* = 3, data points represent median). FSL numbers (Food Safety Laboratory numbers) refer to lab internal designations used for storage and can be found on www.foodmicrobetracker.net; for our populations, these numbers represent frozen samples from the *in vitro* evolutionary experiment; for all strains, these numbers represent frozen individual strains.

The empty control strain was propagated for 7 days, as the population’s colistin MIC remained at 0.5 μg/mL, which is the same MIC as the empty control parent strain’s MIC (Fig. 3A and Fig. S1). For *mcr-*9 populations, we observed daily fluctuations in the highest colistin concentration that populations grew in throughout their 14 day propagation. The *mcr-9* populations consistently grew in colistin concentrations as high as 0.125 μg/mL–0.5 μg/mL but never grew in colistin concentrations that exceeded 1 μg/mL, which represents a concentration that is 2× higher than the *mcr-9* parent strain’s MIC (Fig. 3B). The day 14 *mcr-9* population showed the same colistin MIC as the parent strains (Fig. 3A).

In contrast, the *mcr-3* populations grew in increasing colistin concentrations every day until day 4, when the population was split into two (Fig. 3C); *mcr-3* populations only grew in colistin concentrations as high as 4 μg/mL on day 1 but grew in concentrations as high as 32 μg/mL by day 4 (Fig. 3C). Immediately after the split, *mcr-3* populations from propagation A continued to grow at higher colistin concentrations (64 μg/mL) and reached 128 μg/mL by day 7. In contrast, populations from propagation B maintained growth at 32 μg/mL colistin throughout the rest of the exposure period (Fig. 3C).

To more accurately measure colistin resistance in the evolved populations, we determined MICs at each timepoint using MHII broth, a standard medium for antimicrobial susceptibility testing. The *mcr-3* population colistin MICs of both propagation A and B were similar to or lower than the colistin concentrations for a given population collected during the *in vitro* evolutionary experiment (Fig. 3D). For example, the day 5 and 6 populations from propagation A were collected from cultures that contained 64 μg/mL of colistin, and their population colistin MICs were 64 μg/mL. While the day 5 to 7 populations of propagation B were collected from cultures that contained 32 μg/mL of colistin, the population colistin MICs for the same populations were only 16 μg/mL.

We also assessed MCR protein expression for all populations throughout the *in vitro* evolutionary experiment. As expected, MCR protein expression was not detected for *E. coli* MG1655 empty populations (Fig. S1B). *E. coli* MG1655 *mcr-3* populations showed MCR-3 protein production for populations from each propagation on all days of the experiment (Fig. S2). In contrast, the *E. coli* MG1655 *mcr-9* populations did not show detectable MCR-9 protein production for the duration of the experiment (Fig. S2).

### *E. coli* MG1655 *mcr-3* populations acquire mutations in genes encoding the transcriptional regulator H-NS and enzymes involved in LPS biosynthesis and modification when exposed to colistin

To determine genetic drivers of evolved colistin resistance, we performed whole-genome shotgun sequencing on 12 *mcr-3* populations, including populations collected (i) every day on days 1–4, (ii) every day from each propagation A and B on days 5–7, and (iii) from each propagation A and B on day 14 of the *in vitro* evolutionary experiment, as well as the day 7 and 14 populations for the empty control and *mcr-9* populations, respectively (Fig. 2). On day 7, we only detected two mutations in the empty control population, in comparison to the parent genome; each mutation was in a different intergenic region. In contrast, on day 14, we identified a total of nine mutations in the *mcr-9* population: three mutations which were all located in the same intergenic region and six mutations located in different genomic areas including (i) one intergenic region, (ii) three genes with putative functions, and (iii) two genes with known functions. All mutations in the *mcr-9* populations were detected in <60% of the population, based on read coverage (Table S1). In the *mcr-3* populations from propagation A and B, we identified six and five nonsynonymous mutations, respectively, all fixed to 100% of the population (Fig. 3). In addition, we identified, in these populations, several other mutations that were each found in <30% of the population; these mutations occurred in intergenic regions, pseudogenes, or other genes resulting in amino acid changes (Table S1).

**Fig 4:**
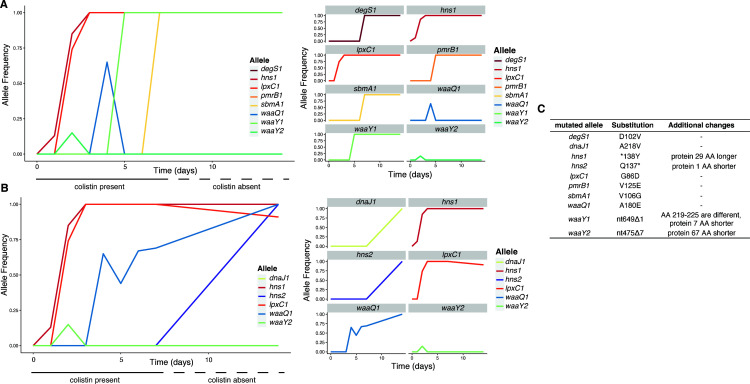
Nonsynonymous mutations acquired by *E. coli* MG1655 *mcr-3* populations. (**A, B**) Changes in allele frequencies of mutated alleles are shown for *E. coli* MG1655 *mcr-3* propagation A (**A**) and propagation B (**B**) in the presence (solid line) and absence (dashed line) of colistin; allele frequencies were determined with breseq (22). (**C**) Mutated alleles and their respective changes are shown for all mutated alleles in the *E. coli* MG1655 *mcr-3* populations with an allele frequency of >30%; the *waaY2* mutation was also included, as another mutation in *waaY* (*waaY1*) was detected in propagation A. All mutated alleles of the populations for *E. coli* MG1655 empty, *mcr-3*, and *mcr-9* can be found in Table S1. * indicates a stop codon; Δ followed by a number indicates a deletion of *n* bases; each resulted in frameshifts.

All nonsynonymous mutations present in 100% of the *mcr-3* populations at any point during the *in vitro* evolution were found in genes involved in LPS biosynthesis and modification, along with one mutation in a transcription factor gene. In the initial 4 days before dividing the population into two separate propagations, the *mcr-3* populations acquired a nonsynonymous mutation in each of *lpxC* and *hns* (named *lpxC1* and *hns1*) by day 2 (see Fig. 4C), followed by a nonsynonymous mutation in *waaQ* (*waaQ1*) on day 4 (Fig. 4A and 4B). These mutated genes encode a UDP-(3-*O*-acyl)-*N*-acetylglucosamine deacetylase (LpxC), which catalyzes the rate-limiting step of lipid A synthesis (18), a histone-like nucleoid structuring protein (H-NS), which directly and indirectly regulates a large regulon (19, 20), and a lipopolysaccharide core heptosyltransferase 3 (WaaQ), which adds the side-branch heptose of the inner core of LPS; this side-branch heptose is a prerequisite for WaaY function (21).

After being split, the *E. coli* MG1655 *mcr-3* populations from each propagation showed distinct trends in their already mutated alleles and in the acquisition of new, additional mutations (Fig. 4A and 4B). For the populations from propagation A, the *lpxC1* and *hns1* mutations were maintained during propagation in the presence and absence of colistin. After day 4, the *waaQ1* mutation was no longer identified in the populations of propagation A. However, a nonsynonymous mutation in *pmrB* (*pmrB1*) and a small deletion within *waaY* (*waaY1*) were first identified on day 5 (Fig. 4A). *pmrB*, also known as *basS*, encodes the sensor kinase PmrB of the PmrAB two-component system, which regulates the expression of *eptA,* a chromosomal lipid A phosphoethanolamine transferase (23). Mutations in *pmrB* are generally associated with spontaneous colistin resistance (24). *waaY* encodes the lipopolysaccharide core heptose(b) kinase, WaaY, which phosphorylates the second heptose unit of the inner core of the core-lipid A molecule (21). We detected two additional mutations in the populations of propagation A on day 7: one nonsynonymous mutation in each *degS* and *sbmA* (i.e., *degS1* and *sbmA1*; Fig. 4A). These genes encode the serine protease DegS, a membrane-anchored protease that senses protein folding stress (25), and the inner membrane transporter SbmA, which transports proline-rich antimicrobial peptides into the cell (26).

In propagation B, the frequency of the *waaQ1* mutation increased from 44% of the population on day 5 to fixation (i.e., identification in 100% of reads) by day 14. We also detected two new mutations in the day 14 population of propagation B (i.e., after colistin selective pressure was removed): *hns2,* which likely reverts the impact of the *hns1* mutation, as it converts the penultimate codon of the WT *hns* gene into a stop codon, and a nonsynonymous *dnaJ1* mutation (Fig. 4B). *dnaJ* encodes the chaperone protein DnaJ, involved in protein translation and folding (27).

### Evolved *E. coli* MG1655 *mcr-3* strains show varying colistin MICs

To determine the contributions of individual mutations to the colistin susceptibility phenotypes in *E. coli* MG1655 *mcr-3* populations observed during the *in vitro* evolutionary experiment, we isolated six individual strains from the collected populations, representing all possible combinations of individual mutations that occurred during propagations A and B. These six strains included three strains obtained while colistin was present and three obtained after colistin pressure was removed. These strains will be referred to as “evolved strains” and are named based on our laboratory’s internal FSL (“Food Safety Laboratory”) numbers (Table 1). We conducted colistin MIC assays on our six evolved strains along with the *E. coli* MG1655 *mcr-3* parent strain (Table 1). We found that all evolved strains had higher colistin MICs than the *mcr-3* parent strain, but colistin MICs varied between strains (Table 1). While each evolved strain had at least a 2× higher MIC than the *mcr-3* parent strain, none of the MICs were as high as those recorded for the populations the evolved strains were collected from (Fig. 3D). This suggests that there likely are population dynamics at play that further increase the colistin MICs of the populations (Fig. 3D).

**TABLE 1:** Colistin MICs of isolated evolved *E. coli* MG1655 *mcr-3* strains^a^

Strain^b^	Isolated from^c^	Nonsynonymous mutations^c^	Colistin MIC (μg/mL)^d^
S14-0047	N/A	N/A	4
S14-0084	Day 2 population	*lpxC1, hns1*	8
S14-0085	Day 4 population	*lpxC1, hns1, waaQ1*	16
S14-0086	Day 14A population	*lpxC1, hns1, pmrB1, waaY1, sbmA1, degS1*	32
S14-0090	Day 14B population	*lpxC1, hns1, hns2, waaQ1, dnaJ1*	8
S14-0091	Day 5A population	*lpxC1, hns1, pmrB1, waaY1*	32
S14-0221	Day 14B population	*hns1, hns2, waaQ1, dnaJ1*	8

^a^ Evolved strains were chosen, such that they represented all possible combinations of individual mutations that occurred during propagation A or B (for details on selection, see the Materials and Methods section).

^b^ Evolved mutants’ strain names are referred to as FSL numbers for simplicity.

^c^ N/A, not applicable, which means strain was not isolated from a population and did not include nonsynonymous mutations.

^d^ Colistin MICs are given as medians of three independent biological replicates.

### *E. coli* MG1655 *mcr-3*-evolved strains have additional lipid A modifications not found in the *mcr-3* parent strain

**Fig 5:**
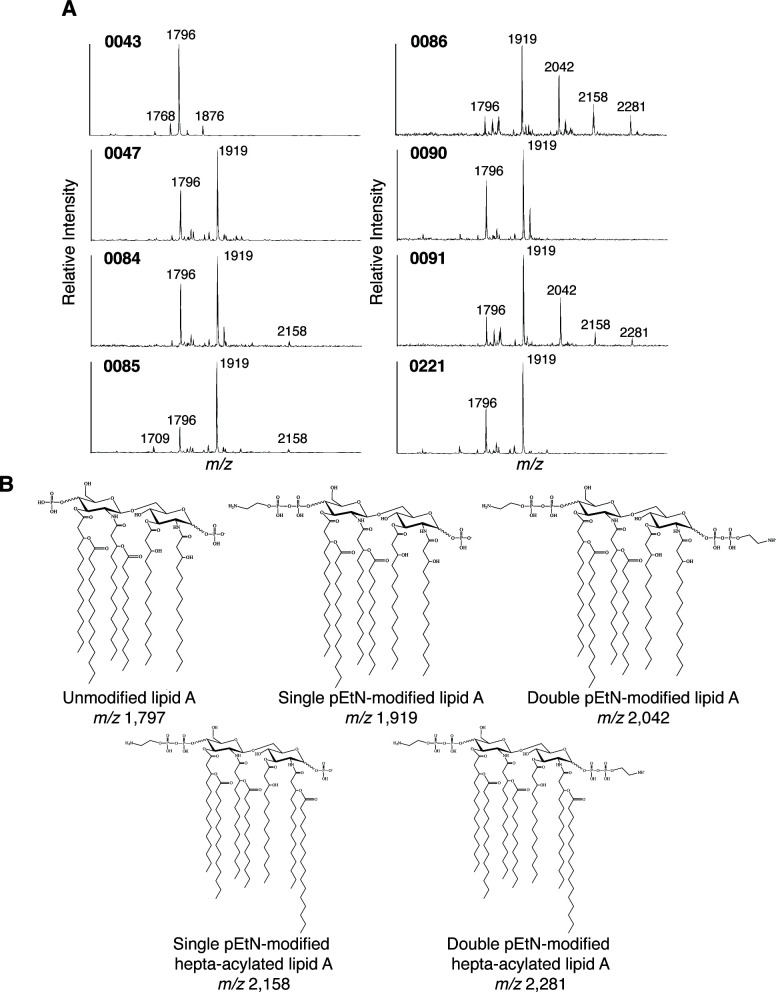
FLAT mass spectra and chemical structure of lipid A of evolved *mcr-3* strains. (**A**) The six *E. coli* MG1655 *mcr-3*-evolved strains (S14-0084–S14-0221), their *mcr-3* parent strain (S14-0047), and empty control strain (S14-0043) show several lipid A signals at *m/z* 1,796, 1,919, 2,024, 2,158, and 2,281. The “S14-” was dropped from the strain names for ease of visualization; the specific mutated genes and a summary of all the lipid A species found in each of the *mcr-3*-evolved strains can be found in Table 2. (**B**) Chemical structures of each of the five major lipid A species detected in our *mcr-3* mutants. The 1,797, 1,919, 2,024, 2,158, and 2,281 signals represent hexa-acylated (WT) lipid A, single pEtN-modified lipid A, hepta-acylated and single pEtN-modified lipid A, double pEtN-modified lipid A, and hepta-acylated and double pEtN-modified lipid A, respectively.

To determine whether mutations located in genes related to lipid A biosynthesis (*lpxC*) and terminal phosphate modification (*pmrB*) altered the structure of lipid A in the evolved strains, we performed fast lipid analysis technique (FLAT) followed by matrix-assisted laser desorption/ionization time-of-flight mass spectrometry (MALDI-TOF MS) on the six evolved *mcr-3* strains, as well as the empty and *mcr-3* parent strains as controls. The ion detected at m/z 1,796 appeared in all strains evaluated, including the empty control and the *mcr-3* parent strain, representing the unmodified WT bis-phosphorylated hexa-acylated lipid A species (Fig. 5A). The ion detected at m/z 1,919 corresponds to lipid A modified with a single pEtN group and is observed in all *mcr-3* strains (Fig. 5A).

Interestingly, several evolved *mcr-3* strains exhibit additional lipid A-modified species absent in the *mcr-3* parent strain, including (i) ions at *m/z* 2,042 indicating double pEtN-modified lipid A, (ii) ions at *m/z* 2,158 indicating hepta-acylated, single pEtN-modified lipid A (through the addition of a palmitoyl fatty acid chain), and (iii) ions at *m/z* 2,281 indicating hepta-acylated, double pEtN-modified lipid A (Fig. 5). Evolved strains S14-0090 (with *lpxC1*, *hns1, hns2, waaQ1, dnaJ1* mutations) and -0221 (with *hns1, hns2, waaQ1, dnaJ1* mutations) show the WT and pEtN-modified lipid A species, while strains S14-0084 (*lpxC1, hns1* mutations) and -0085 (*lpxC1, hns1, waaQ1* mutations) additionally show hepta-acylated lipid A. Strains S14-0086 (*lpxC1, hns1, pmrB1, waaY1, smbA1, degS1* mutations) and -0091 (*lpxC1, hns1, pmrB1, waaY1* mutations) show all previously mentioned modified lipid A species (Table 2; Fig. 5).

**TABLE 2:** Lipid A species detected for evolved *E. coli* MG1655 *mcr-3* strains using FLAT

Strain^a^	Nonsynonymous mutations^b^	Detected lipid A species^c^				
		Unmodified	Single pEtN modification	Double pEtN modification	Single pEtN modification + hepta-acylation	Double pEtN modification + hepta-acylation
S14-0043	N/A	x				
S14-0047	N/A	x	x			
S14-0084	*lpxC1, hns1*	x	x		x	
S14-0085	*lpxC1, hns1, waaQ1*	x	x		x	
S14-0086	*lpxC1, hns1, pmrB1, waaY1, sbmA1, degS1*	x	x	x	x	x
S14-0090	*lpxC1, hns1, hns2, waaQ1, dnaJ1*	x	x			
S14-0091	*lpxC1, hns1, pmrB1, waaY1*	x	x	x	x	x
S14-0221	*hns1, hns2, waaQ1, dnaJ1*	x	x			

^a^ Evolved mutants’ strain names are referred to as FSL numbers for simplicity.

^b^ N/A, not applicable, meaning that strain did not include nonsynonymous mutations.

^c^ Lipid A species that were detected using FLAT and MALDI-TOF MS (Fig. 5) are indicated with an x.

### Evolved *E. coli* MG1655 *mcr-3* strains differ in their MCR-3 protein levels and context-dependent fitness

**Fig 6:**
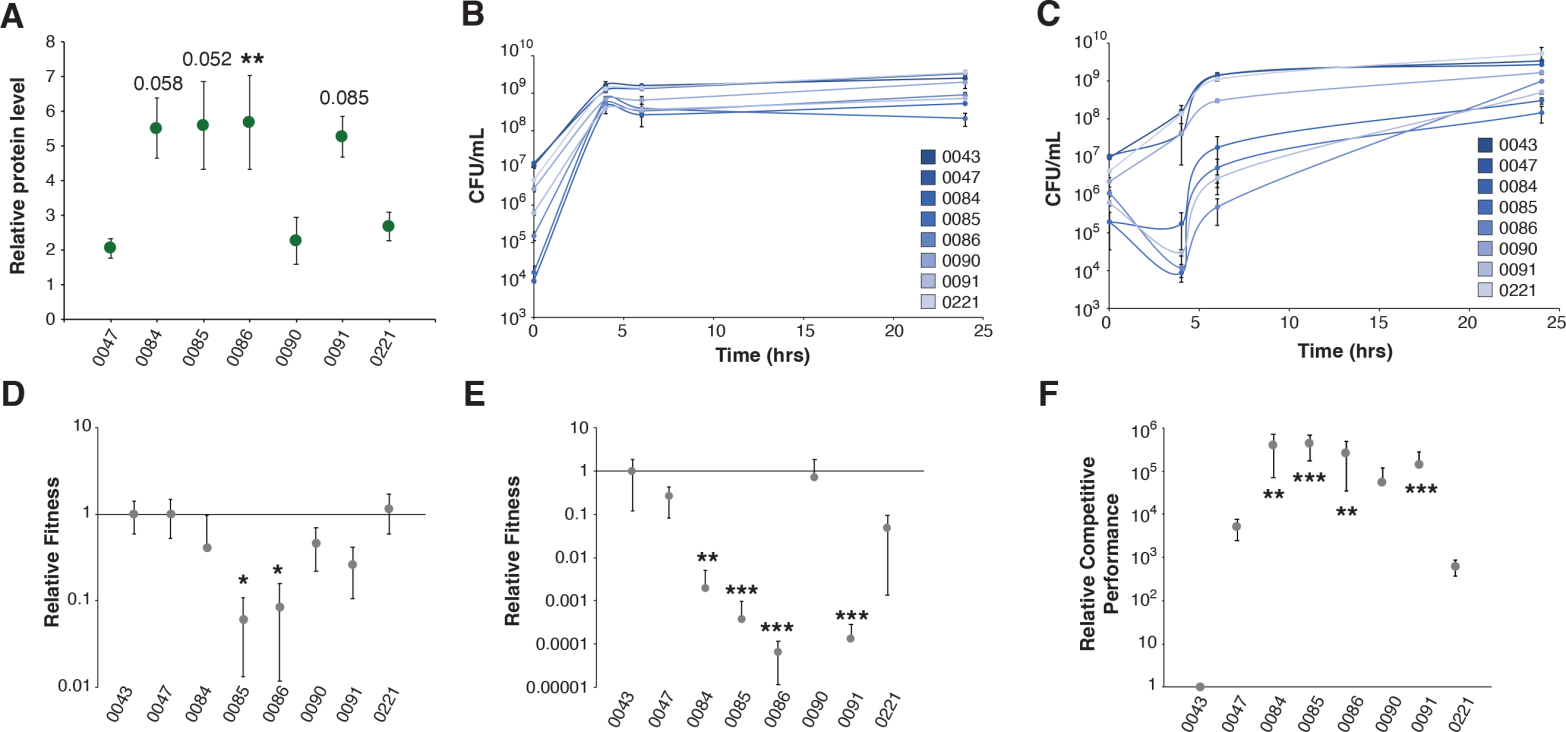
Relative MCR-3 levels, bacterial growth, and context-dependent relative fitness of evolved *E. coli* MG1655 *mcr-3* strains. (**A**) The relative MCR-3 protein levels of evolved *E. coli* MG1655 *mcr-3* strains (S14-0084–S14-0221) and their *mcr-3* parent strain (S14-0047) were determined after strains were grown in BHI for 6 h. The “S14-” was dropped from strain names for ease of visualization; the specific mutated genes found in each of the *mcr-3*-evolved strains are listed in Table 1. Anti-MYC antibodies were used to detect MYC-tagged MCR-3, SDS-PAGE gels, and Western blots used to determine relative MCR-3 levels are shown in Fig. S3. (**B, C**) The bacterial growth of evolved *mcr-3* strains grown in BHI (**B**) or BHI with 5% (vol/vol) human serum (**C**) was assessed over 24 h. (**D, E, F**) The relative fitness/competitive performance of all evolved strains, and their parent strain, was determined after 6 h competitions with competitor strain *E. coli* MG1655 pUC19 in BHI (**D**), BHI with 5% (vol/vol) human serum (**E**), or BHI with 2 μg/mL of colistin (**F**). Relative fitness/competitive performance values are shown relative to the *E. coli* MG1655 empty strain’s fitness values. For all panels: data points represent means (*n* = 3), and error bars represent the standard error of the mean. One-way ANOVAs with *post hoc* Dunnett’s test in comparison to the *E. coli* MG1655 *mcr-3* parent strain, S14-0047, were used to identify significant differences relative to the *E. coli* MG1655 *mcr-3* parent strain (to fulfill the criteria of normality, data for assays performed in the presence of colistin or human serum were log-transformed); *, *P* < 0.05; **, *P* < 0.01; ***, *P* < 0.01.

Since the evolved *E. coli* MG1655 *mcr-3* strains showed distinct colistin MICs and carried mutations in genes linked to inner core lipid A synthesis (*lpxC, waaQ, waaY*), terminal phosphate modification (*pmrB*), and *hns*—a global transcriptional regulator (28), we hypothesized that these mutations impact (i) MCR-3 protein levels, (ii) context-dependent growth, and (iii) relative fitness. To test these hypotheses, MCR-3 protein levels were assessed after the six evolved *mcr-3* strains were grown in BHI media for 6 h. While, as expected, MCR-3 expression was detected in all evolved *mcr-3* strains, as well as their *mcr-3* parent strain (Fig. S3), the six evolved strains seem to represent two groups with regard to relative MCR-3 protein levels, including one group with MCR-3 levels comparable to the parent strain (S14-0090, -0221) and another group with significantly (*P* < 0.1) higher MCR-3 levels compared to the parent strain (S14-0084, -0085, -0086, and -0091) (Fig. 6A).

We also compared bacterial growth of the six evolved *mcr-3* strains to the parent strain in both BHI and BHI supplemented with human serum (Fig. 6B and 6C). Experiments with non-heat-inactivated human serum were carried out to more closely mimic host environments and to assess possible complement system interactions with LPS (29, 30). When grown in BHI, all tested strains showed similar growth patterns (Fig. 6B). While the starting bacterial numbers of all strains fall within a wide range (10^4^–10^7^ CFU/mL), likely due to variations in the inoculum (Fig. 6B), all strains had similar cell numbers (10^8^–10^9^ CFU/mL; Fig. 6B) after 4 and 24 h of growth. When grown in BHI supplemented with human serum, two distinct growth phenotypes occurred (Fig. 6C). While evolved strains S14-0090 and -0221 showed similar growth dynamics and bacterial numbers as the empty control and the *mcr-3* parent strain, evolved strains S14-0084, -0085, -0086, and -0091 showed a steep decline in cell numbers within the first 4 h of the experiment (10^5^–10^6^ to 10^4^ CFU/mL decline), followed by subsequent growth that resulted in bacterial numbers similar to those of the *mcr-3* parent strain after 24 h (Fig. 6C).

To better understand whether the acquired mutations provided competitive advantages or disadvantages under different growth conditions, we performed competition experiments for the *mcr-3*-evolved and parent strains in three different conditions: (i) BHI, (ii) BHI supplemented with human serum, and (iii) BHI supplemented with colistin. We calculated fitness (assays in BHI and BHI supplemented with human serum) and competitive performance (assay under colistin selection) as the ratio of surviving *mcr-3* strains to surviving competitor strains (*E. coli* MG1655 pUC19), normalized by their bacterial numbers at the beginning of competition (13); more details can be found in the Materials and Methods section. All fitness and competitive performance values were converted to relative values, based on the *E. coli* MG1655 empty strain’s fitness or competitive performance value in each experimental condition. Similar to growth assay results, when grown in BHI without additional supplements, evolved strains S14-0084, -0090, -0091, and -0221 showed relative fitness values comparable to the *mcr-3* parent strain, while S14-0084 and -0085 showed significantly lower fitness; these two strains were recovered at approximately 10-fold lower levels (*P* < 0.05) than the competitor strain (Fig. 6D). Generally, the relative fitness values of the six evolved *mcr-3* strains in the presence of human serum mirror the trends seen in the growth assessment performed in the presence of human serum. While the relative fitness values of evolved strains S14-0090 and -0221 do not significantly differ from their *mcr-3* parent strain, strains S14-0084, -0085, -0086, and -0091 have significantly (*P* < 0.01) reduced relative fitness values in the presence of human serum; fitness values of these four strains are 10–100 times lower than those of the *mcr-3* parent (Fig. 6E). Under colistin selection, all strains with *mcr-3*, including the *mcr-3* parent strain, showed competitive advantages when compared to the empty control strain, with relative competitive performance values ranging from 1,000 to over 100,000 (Fig. 6F). However, when comparing the evolved *mcr-3* strains to their parent strain, strains S14-0090 and -0221 do not have significantly different relative competitive performance values (Fig. 6F). In contrast, strains S14-0084, -0085, -0086, and -0091 have significantly (*P* < 0.01) higher relative competitive performance values than the *mcr-3* parent strain under colistin selection.

### Mutations in *lpxC* and *hns* significantly impact several phenotypes of evolved *E. coli* MG1655 *mcr-3* strains

To quantify the phenotypic impact of individual mutations in evolved *mcr-3* strains, we created linear regression models assessing the effects of the individual mutations on MCR-3 protein level, growth in BHI, growth in the presence of human serum, fitness in BHI, competitive performance under colistin selection, and fitness in the presence of human serum. Because some of the mutations occurred simultaneously, the *waaY1* and *pmrB1* mutations were combined into a single predictor, and so were the *sbmA1* and *degS1* mutations. The final linear regression models (Table 3) include a constant and varying numbers of mutations as predictors. The constant represents the estimated value of each outcome when all predictors are zero; essentially, the predicted phenotype of the *mcr-3* parent strain without any mutations. Each model has a different number of predictors because the final models represent the model with the best fit based on the Akaike Information Criterion. When a predictor is significant, it indicates that the mutation is linked to measurable changes in the phenotype. Each regression coefficient indicates the expected phenotypic change per unit change in the predictor.

**TABLE 3:** Summary statistics for linear regression models to predict the impact of individual mutations on selected phenotypes^*c*^

	MCR-3 protein level	Bacterial growth (BHI)	Bacterial growth (HS)^*a*^	Fitness (BHI)	Competitive performance (colistin)^*a*^	Fitness (HS)^*a*^
Constant	2.334***	1.156E09***	7.605***	1.089***	3.674***	−0.624°
*lpxC1*	–	−6.859E08***	−0.75	−0.833***	1.793***	–
*hns1*	3.168***	–	−2.543***	–	–	−2.755***
*waaQ1*	–	2.078E08*	−0.502	–	–	−0.573
*dnaJ1*	–	–	0.814	–	−0.933***	–
*waaY1, pmrB1* ^*b*^	–	–	–	–	−0.382	−0.894°
*sbmA1, degS1* ^*b*^	–	–	–	–	–	–
R-squared	0.616	0.758	0.922	0.558	0.853	0.85
Adjusted R-squared	0.596	0.731	0.902	0.535	0.827	0.8234
F-statistic (DF)	30.43 (1, 19)	28.21 (2, 18)	47.12 (4, 16)	23.99 (1, 19)	32.91 (3, 17)	32.2 (3,17)
*P*-value	2.54E-05	2.83E-06	1.18E-08	1.00E-04	2.66E-07	3.11E-07

^*a*^ Log-transformed data were used to run the linear models for bacterial growth (HS), competitive performance (colistin), and fitness (HS), because the untransformed data did not follow the normal distribution. HS, human serum.

^*b*^ Collinear variables were combined as one predictor.

^*c*^ –, predictor variable was not included in the final model. *P*-values for individual predictors are shown as follows: °, *P* < 0.1; *, *P* < 0.05; **P < 0.01; ***P < 0.001.

The linear regression models for the evolved *mcr-3* strains and their associated phenotypes identified the constant as a significant (*P* < 0.05) predictor of all assessed phenotypes except for relative fitness in human serum (Table 3), suggesting that the presence of the functional MCR-3 protein significantly impacts most of the assessed phenotypes. The predictor that includes the *sbmA1* and *degS1* mutations was not included in any of the models, as it did not seem to improve overall model fit, suggesting that these two mutations may impact phenotypes not assessed here or may only have limited impacts on the measured phenotypes. While the predictor representing both the *waaY1* and *pmrB1* mutations was included in the models for relative fitness in the presence of human serum and competitive performance under colistin selection, it is not a significant (*P* < 0.05) predictor of either phenotype (Table 3), suggesting neither mutation significantly impacts either phenotypic outcome. The *dnaJ1* mutation was found to be a significant (*P* < 0.001) negative predictor of competitive performance under colistin selection and was also included as a predictor, albeit not significant, for bacterial growth in the presence of human serum (Table 3). This suggests that the *dnaJ1* mutation likely reduces the fitness of *mcr-3* strains in the presence of colistin. While the *waaQ1* mutation was included as a predictor for bacterial growth in BHI and growth or fitness in the presence of human serum, it only significantly (*P* < 0.05) positively predicted growth in BHI (Table 3). This suggests that the *waaQ1* mutation likely increases growth of *mcr-3* strains when grown in BHI. The *hns1* mutation was included as a significant (*P* < 0.001) predictor in the models for MCR-3 protein level as well as growth and fitness in the presence of human serum (Table 3). This suggests that the *hns1* mutation likely increases MCR-3 protein levels but decreases growth and fitness in the presence of human serum for *mcr-3* strains. Lastly, the *lpxC1* mutation was not a significant predictor of growth in the presence of human serum, but a significant (*P* < 0.001) negative predictor of growth and fitness in BHI, as well as a significant (*P* < 0.001) positive predictor of competitive performance under colistin selection. This suggests that the *lpxC1* mutation could lead to a decrease in bacterial growth and fitness of *mcr-3* strains in BHI but increases the competitive performance of *mcr-3* strains under colistin selection.

### MCR-3 protein levels correlated with context-dependent bacterial growth and fitness

**Fig 7:**
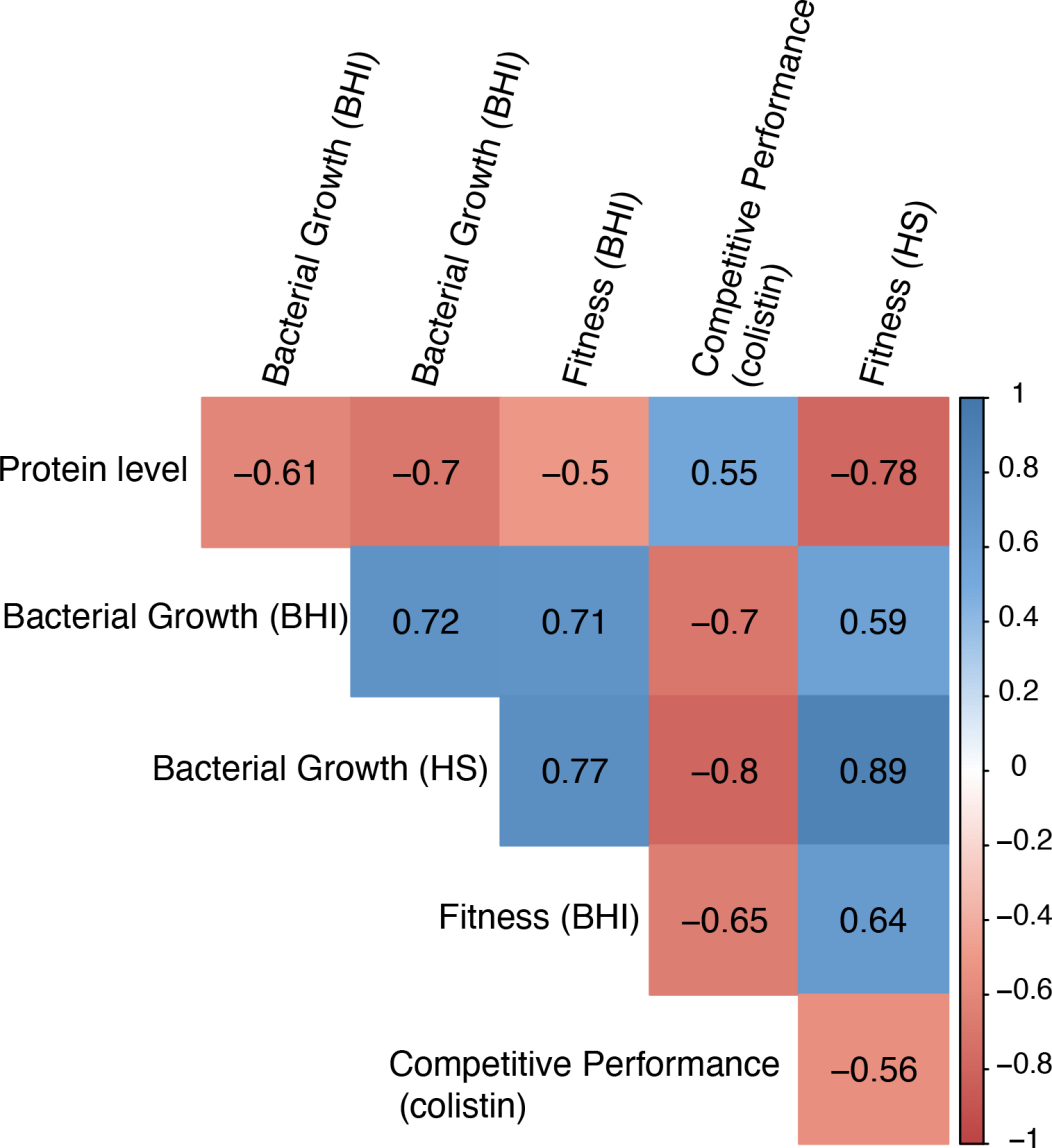
Correlations between phenotypic outcomes of evolved *E. coli* MG1655 *mcr-3* strains. Pearson’s correlation coefficients were calculated for all tested phenotypes, except for colistin MIC values, and are shown in the correlation matrix if *P* < 0.05 using the corrplot package (31); all correlations were significant. Log-transformed values of bacterial growth in the presence of human serum, relative competitive performance under colistin selection, and relative fitness in the presence of human serum were used. HS, human serum.

To determine whether any of the assessed phenotypes of the six evolved *mcr-3* strains are correlated, we performed a correlation analysis. As Fig. 7 shows, all assessed phenotypic outcomes are significantly correlated (*P* < 0.05), with Pearson’s correlation coefficients ranging from 0.5 to 0.89 (including both positive and negative coefficients). While it is not surprising that growth and fitness data assessed in BHI are correlated with each other and correlated with growth and fitness data assessed in the presence of human serum, it is interesting that competitive performance under colistin selection is negatively correlated with those four phenotypes (Fig. 7). Additionally, MCR-3 protein levels are positively correlated with the competitive performance under colistin selection but negatively correlated with growth and fitness in both BHI and in the presence of human serum (Fig. 7). Overall, our data indicate that higher MCR-3 protein levels are correlated with increased competitive performance under colistin selection but lower fitness in the absence of colistin.

### Nonsynonymous mutations in *lpxC* and *hns* are very rare in clinical and environmental *E. coli* isolates

Since the nonsynonymous mutations in *lpxC* and *hns* significantly impacted several phenotypes of the evolved *mcr-3* strains, particularly growth and fitness outcomes (Table 3), we determined whether *lpxC* and *hns* single nucleotide polymorphisms (SNPs) and nonsynonymous mutations are common in WT *E. coli* strains. To assess how common these mutations are, we randomly selected genome assemblies of 2,000 *E. coli* isolates from the NCBI Pathogen Detection database for analysis (Table S2). Because one assembly was not available, our analysis included 1,999 isolates in total. The randomly selected isolates were highly diverse, as they represented 507 different sequence types (Table S2). In comparison to the *E. coli* MG1655 *lpxC* and *hns* gene and protein sequences, 65.9% and 73.8% of isolates had SNPs in their *lpxC* and *hns* genes, respectively, but only 5.05% (101/1,999) and 9.6% (191/1,999) of all isolates had nonsynonymous mutations in LpxC and H-NS, respectively. Among the 55 isolates within our data set that carried *mcr* variants, none showed nonsynonymous mutations in either *lpxC* or *hns* in comparison to the *E. coli* MG1655 sequences.

## DISCUSSION

The spread of *mcr* genes represents a major public health concern, particularly amid the increase in MDR bacteria. To determine how bacteria carrying relatively understudied *mcr* variants adapt to colistin selective pressure, we exposed *E. coli* MG1655 carrying single copies of *mcr-3* or *mcr-9* under their native promoters to colistin and assessed changes in genotypes and phenotypes for populations, as well as for individual evolved strains. Overall, our data indicate that the *E. coli* MG1655 strain that expressed MCR-3 adapted to colistin selective pressure through nonsynonymous mutations and increased colistin MICs. We found that mutations in LPS biosynthesis and modification genes appear to confer higher colistin MICs in *E. coli* MG1655 *mcr-3* strains. Mutations in LpxC were found to increase the competitive performance of *E. coli* MG1655 *mcr-3* strains under colistin selection but may impose fitness costs in the absence of colistin. Lastly, H-NS seems to regulate expression of LPS modification genes and *mcr-3* within the context of evolved colistin resistance.

### *E. coli* strains expressing MCR-3 proteins adapt to colistin selective pressure

Even though we exposed three different *E. coli* MG1655 strains to colistin for several days, only the strain expressing functional MCR-3 adapted to colistin selective pressure by nonsynonymous mutations and increased colistin MICs. The lack of an increased colistin MIC in the *E. coli* MG1655 *mcr-9* parent strain during our *in vitro* evolutionary experiment is likely due, at least in part, to undetectable MCR-9 protein expression. There is some evidence that this lack of expression may be due to repression of *mcr-9* transcription facilitated by an inverted repeat region upstream of the native *mcr-9* promoter (32). The lack of *mcr-9* expression we observed is also consistent with other studies that have shown that *mcr-9* expression is low in clinical strains (29) and that natural *mcr-9*-carrying strains are often susceptible to colistin (16, 30). However, it also has been reported that *mcr-9*-carrying *Enterobacter* spp., which did not show phenotypic colistin resistance when grown in MHII (the standard media used for MIC assays), showed resistance when grown in BHI (33). One study also showed that *mcr-9*-carrying *E. coli* modifies its lipid A with pEtN when exposed to subinhibitory concentrations of colistin (17), suggesting *mcr-9* is expressed. Our finding that the colistin MIC of the *mcr-9* strain did not increase was thus surprising, as the strain was (i) grown in BHI and (ii) exposed to subinhibitory concentrations of colistin. This finding further supports that regulation of *mcr-9* and its ability to confer colistin resistance are complex (17).

Interestingly, we also did not find any evidence of colistin adaptations or an increased colistin MIC in the WT strain with an “empty” transposon, which was also included in the *in vitro* evolutionary experiment. We speculate that one reason for this observation may be that mutations that could arise during an *in vitro* evolutionary experiment could be outcompeted by their parent strains, likely due to fitness costs associated with these mutations. This explanation could also be relevant for the lack of changes in colistin susceptibility in the *mcr-9* strain. Possible fitness costs carrying mutations that could result in colistin resistance in the empty or *mcr-9* strains include chromosomal mutations that result in expression of EptA or MCR-9, respectively, and subsequent phosphoethanolamine modification of the 1-phosphate of lipid A. Both modification of the 1-phosphate of lipid A (13) and chromosomal mutations resulting in colistin resistance (34) have previously been reported to be associated with higher cell viability defects or fitness costs.

In contrast to the empty and *mcr-*9 strains, the *E. coli* MG1655 *mcr-3* strain adapted to colistin selective pressure, as indicated by the acquisition of several nonsynonymous mutations and increases in population colistin MICs. This is consistent with a previous study (11) that showed that strains expressing *mcr-1* display higher evolvability under colistin selective pressure than non-*mcr-1* control strains. Characterization of six *mcr-3* strains that evolved during the *in vitro* evolutionary experiment showed that these strains represented two distinct groups. Isolates referred to as group 1 were (i) obtained from both propagations A and B during colistin exposure, (ii) consisted of strains S14-0084, -0085, -0086, and -0091, and (iii) had phenotypes distinct from the *mcr-3* parent strain. Isolates referred to as group 2 (i.e., S14-0090 and -0221) were isolated from propagation B after colistin exposure and had phenotypes similar to the *mcr-3* parent strain, including an MIC that was only 2× higher as compared to the parent strain. Group 1 isolates had (i) 4–8× higher colistin MICs, (ii) additional lipid A modifications, (iii) significantly higher MCR-3 protein levels, and (iv) significantly increased competitive performance under colistin selection, as well as (v) significantly reduced bacterial growth in the presence of human serum and (vi) significantly reduced fitness in standard media and in the presence of human serum in comparison to the *mcr-3* parent strain. Our findings indicate that distinct evolutionary trajectories can occur over the course of antibiotic exposure, consistent with previous studies (11, 35).

Interestingly, fitness in media without colistin and the competitive performance under colistin selection were negatively correlated in the evolved strains. Opposing relationships between the acquisition of AMR factors and fitness have been reported previously (36), especially for colistin resistance (5, 13). Due to the negative relationship between AMR and bacterial fitness, it has been hypothesized that acquired mutations may revert after the antibiotic pressure is removed, or that additional mutations may be acquired in order to reduce the fitness costs of mutations that confer AMR (37). We observed this in *mcr-3*-evolved strains within propagation B when H-NS reverted to its WT structure through the *hns2* mutation and *lpxC1*’s allele frequency reduced to 91%, which gave rise to evolved strains with similar phenotypes as the *mcr-3* parent strain. However, we also saw an alternative evolutionary trajectory, where populations in propagation A retained all acquired mutations and did not acquire any new mutations in the absence of colistin selective pressure. More specifically, we did not find any evidence for adaptations that reduced the negative fitness consequences associated with colistin resistance; however, future experiments, such as continued propagation in subinhibitory MIC concentrations (or propagation under different conditions), may be valuable to further explore the possible emergence of these types of mutations.

Overall, our results indicate that the expression of functional *mcr* variants facilitates the accumulation of nonsynonymous mutations, which seem to impact several phenotypes and result in higher colistin MICs. While colistin MICs higher than the breakpoint, that is, ≥4 µg/mL based on CLSI guidelines, may not be clinically relevant, our findings suggest that *mcr-*expressing strains adapt to colistin selective pressure more readily than strains that do not express *mcr* variants. This may especially be relevant to bacteria carrying *mcr*-variants which have colistin MICs right below the breakpoint. Additionally, our findings suggest that accumulated mutations can have distinct evolutionary trajectories once colistin selective pressure is removed—mutations can either be maintained or be reverted, indicating that the interplay of all acquired mutations might impact their fate in the absence of antibiotic pressure.

### Mutations in LPS biosynthesis and modification genes appear to confer higher colistin MICs in *E. coli* MG1655 *mcr-3* strains

**Fig 8:**
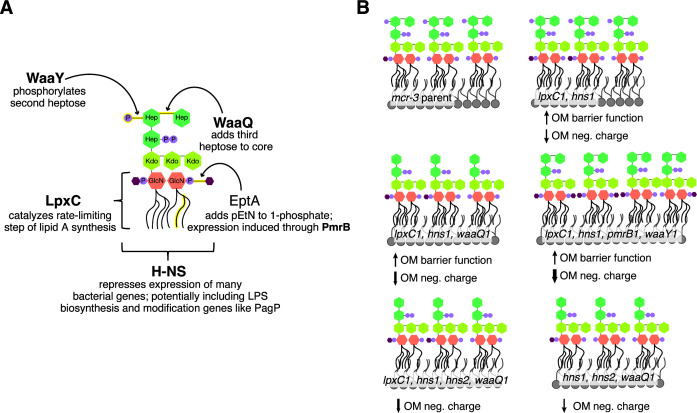
LPS structure and enzymatic functions of LPS biosynthesis and modification enzymes identified to be mutated in the *in vitro* evolutionary experiment. (**A**) The general structure of LPS of *E. coli* is shown; blue hexagons represent the O-antigen, “Hep” represents heptose residues, “Kdo” represents 3-deoxy-D-manno-octulosonic acid, and “GlcN” represents glucosamine. Arrows indicate how WT versions of individual enzymes found to be mutated during the *in vitro* evolutionary experiment impact the structure of lipid A. (**B**) Lipid A-inner core structures of described *mcr-3* mutants and their proposed impacts on the outer membrane (OM) barrier function and negative charge, in comparison to the *mcr-3* parent strain in the context of the outer membrane.

*E. coli* MG1655 *mcr-3*-evolved strains primarily adapted to colistin exposure through mutations in genes involved in LPS biosynthesis and modification, though mutations in other genes were also detected. While mutations in *waaQ, waaY, pmrB, degS, dnaJ,* and *sbmA* showed limited statistical support for their phenotypic impact, they may still affect the assessed phenotypes and specifically colistin resistance. WaaQ and WaaY are both involved in LPS inner core synthesis (Fig. 8A) (21). WaaQ attaches the side-branch heptose to the inner core, and WaaY phosphorylates the second core heptose unit (21). It has been shown that WaaY’s ability to phosphorylate the second heptose is reduced in the absence of the side-branch heptose residue when WaaQ is non-functional (38). Each mutation could result in a lower number of phosphates on LPS. This could reduce the negative charge of the outer membrane, thus decreasing bacterial susceptibility to colistin, consistent with the higher colistin MICs of the evolved strains with *waaQ1* and *waaY1* mutations (Fig. 8B). Our findings are consistent with previous data (39) that showed that increased *waaY* expression, through inhibition of its regulator MarR, increased the negative charge of the outer membrane as well as colistin susceptibility. While neither the *waaQ1* nor the *waaY1* mutation was identified as a positive predictor of competitive performance under colistin selection in the linear regression models, these mutations may still impact colistin resistance.

PmrB encodes a sensor kinase that results in the upregulation of phosphoethanolamine transferase EptA (Fig. 8) (1, 23). Mutations in *pmrB* have been shown to result in increased colistin resistance in several studies; however, the V125E mutation detected here has not been identified before. This mutation occurs within the HAMP (histidine kinases, adenylyl cyclases, methyl-accepting proteins, and phosphatases) domain, which regulates PmrA phosphorylation, resulting in PmrA activation. Activation of PmrA induces *eptA* expression, leading to pEtN modification of lipid A, which decreases the negative charge of the outer membrane (Fig. 8B) and results in colistin resistance (40, 41). In our experiments, evolved strains that encode the *pmrB1* allele show double pEtN-modified lipid A, supporting that the *pmrB1* mutation results in upregulation of EptA (13). Despite the biological plausibility, the *pmrB1* mutation was not identified as a significant predictor of any of the assessed phenotypes in the linear regression models; further characterization of this mutant allele may thus be warranted.

DegS and DnaJ are involved in protein translation and folding—DegS senses protein folding stress in the periplasm (25), and DnaJ folds proteins in the cytoplasm (27). While we have no direct evidence that the *dnaJ1* mutation alters protein function, it was identified as a significant negative predictor of competitive performance under colistin selection, suggesting that this mutation negatively impacts DnaJ’s folding ability. In contrast, the *degS1* mutation was not identified as a predictor of any examined phenotype; however, it is possible that the DegS mutation could impact DegS’s ability to detect misfolded proteins in the periplasm, which may impact bacterial growth. Lastly, the inner membrane transporter SbmA is known to transport proline-rich antimicrobial peptides into the cell (26); mutations in *sbmA* were previously detected in *Klebsiella pneumoniae* evolution experiments in the presence of colistin (35). Identification of *sbmA* mutations across two studies could indicate a role of SbmA in colistin resistance.

While we have found functional support that mutations in the genes discussed in this section could impact relevant bacterial phenotypes, only one of the discussed mutations had statistical support for impacting context-dependent bacterial fitness. It is likely that the impacts of the individual mutations are hard to pinpoint within the context of these experiments, as we are only examining a small number of evolved strains which almost all carry mutations in *lpxC* and *hns*, which had more pronounced phenotypic effects.

### LpxC mutation increases the competitive performance of *E. coli* MG1655 *mcr-3* under colistin selection, but imposes fitness costs in the absence of colistin

The nonsynonymous mutation in LpxC was one of the two mutations with major effects on bacterial phenotypes; this mutation was also detected earliest during the *in vitro* evolutionary experiment. The mutation occurs within an α-helix, turning glycine into aspartic acid, which could impact protein folding and function. While it is possible that the *lpxC1* mutation renders LpxC non-functional, resulting in complete loss of LPS, which is a strategy utilized by colistin-resistant *A. baumannii* (42), it is likely that the mutated LpxC is only functionally impaired, as we were able to isolate lipid A from all evolved *mcr-3* strains.

As LpxC catalyzes the rate-limiting step of lipid A synthesis (Fig. 8A) (18), we hypothesized that impaired LpxC function could reduce lipid A production rates, thus decreasing bacterial growth rates as supported by the *lpxC1* mutation being identified as a significant negative predictor of bacterial growth and fitness in standard media. Impaired LpxC function may reduce lipid A production while enabling MCR-3 to pEtN-modify a greater proportion of lipid A, thereby neutralizing the outer membrane’s negative charge (Fig. 8B). This is supported by the *lpxC1* mutation being identified as a significant positive predictor of competitive performance under colistin selection. Mutations in LpxC have previously been detected during exposure to increasing colistin concentrations in biofilm populations of *K. pneumoniae* (35) and in *E. coli* carrying a natural *mcr-1*-containing plasmid (11), further indicating a role of mutated LpxC in colistin resistance. However, even though Jangir et al. (11) and we found that SNPs in *lpxC* are common in natural *E. coli* isolates, we found that these SNPs rarely result in nonsynonymous mutations. Overall, our results imply that while mutations in LpxC may decrease bacterial susceptibility to colistin, the significant, negative impacts on bacterial fitness and bacterial growth might prevent the establishment of LpxC mutations in natural *E. coli* isolates.

### H-NS appears to regulate expression of LPS modification genes and *mcr-3*

The other nonsynonymous mutation with a major effect on bacterial phenotypes occurred in H-NS. The detected *hns1* mutation changes the stop codon into an amino acid, which results in the translation of 29 additional amino acids; this mutation was detected after the 2nd day of the experiment. Interestingly, a second mutation in *hns*, *hns2*, was detected in propagation B after the populations were grown in the absence of colistin for 7 days. This mutation turns the penultimate codon (based on the WT *hns* allele) into a stop codon, removing the additional amino acids added by *hns1* and likely restoring normal H-NS function. Based on the predicted protein structure of H-NS, it is probable that the *hns1* mutation impacts the C-terminal located DNA-binding domain (19), suggesting that this mutation impairs the DNA-binding function of H-NS and leads to de-repression of the H-NS regulon, which potentially includes enzymes involved in LPS biosynthesis (Fig. 8A) (20). Consistent with this, hepta-acylated lipid A species were only detected in evolved *mcr-3* strains with the *hns1* mutation but not in strains with the *hns2* mutation or the *hns* WT allele. Hepta-acylated lipid A may improve the barrier function of the outer membrane (Fig. 8B) (23). Because these structures of hepta-acylated lipid A are formed through palmitoyl fatty acid addition to lipid A mediated by palmitoyltransferase PagP (43), it is tempting to speculate that H-NS might directly or indirectly regulate *pagP* expression.

We also identified H-NS as a potential regulator of *mcr-3* expression, as *hns1* was the only significant positive predictor of MCR-3 protein levels. In line with this, a previous study (44) found that a second copy of *eptA* was upregulated in an *hns* deletion mutant in *A. baumannii*, which resulted in decreased colistin susceptibility. Because H-NS has been shown to regulate foreign DNA in *Salmonella* (45), it is possible that H-NS can interact with the exogenous *mcr-3* promoter to regulate *mcr-3* expression. H-NS-mediated regulation of *mcr* expression could explain why fitness costs are oftentimes negligible in natural, *mcr-*carrying bacteria (5), which would explain why nonsynonymous mutations in H-NS were rare in natural *E. coli* isolates.

The *hns1* mutation was also identified as a significant negative predictor of both growth and fitness in the presence of human serum. Human serum has been used to mimic host environments (46) and contains components from the complement system that may interact with LPS to inactivate pathogens (47). All evolved *mcr-3* strains with the *hns1* mutation produce a lipid A species that is hepta-acylated and pEtN-modified and not produced by the *mcr-3* parent strain. Although hepta-acylated lipid A has been shown to reduce the Toll-like receptor 4 (TLR4)-mediated inflammatory response as an adaptation to host environments (48), a different study (49) showed that human TLR4 signaling was increased when stimulated with pEtN-modified tetra- or penta-acylated lipid A, as compared to unmodified tetra- or penta-acylated lipid A. This suggests that acyl chain and phosphate modification numbers in lipid A could directly impact the host immune system and could explain why strains with the *hns1* mutation were less fit in the presence of human serum in comparison to their *mcr-3* parent strain.

Altogether, our results indicate that H-NS may directly or indirectly regulate the expression of various LPS modification enzymes across bacterial species, specifically phosphoethanolamine transferases like *eptA* and *mcr*. Combined, the LPS modifications mediated by PagP and MCR may impact bacterial susceptibility to colistin and detection by the immune system, further expanding the broad role H-NS appears to play in regulating genes important in host-pathogen interactions of *Salmonella*, *E. coli* (28), and other gram-negative pathogens (50).

### Limitations

This study has several limitations that should be acknowledged. While *E. coli* MG1655 is useful due to its easy genetic manipulability and accessibility of its annotated genome, it does not produce an O-antigen (51). While lipid A appears to be more important for interactions with colistin (6), the lack of an O-antigen likely impacts bacterial phenotypes in the presence of human serum and could result in evolutionary trajectories during colistin exposure that are distinct from clinical strains. Additionally, the colistin concentrations used here are higher than the colistin plasma concentrations in human patients (10). We used higher colistin concentrations here to exert increased selective pressure and to result in faster outcomes in the laboratory. Lastly, in real-world conditions, bacteria can acquire resistance mechanisms through horizontal gene transfer to adapt to colistin selective pressure, something that is difficult to simulate in a laboratory experiment.

### Conclusion

The goal of this study was to understand whether and how bacteria carrying *mcr* variants other than *mcr-1* evolve when exposed to colistin selective pressure. Despite the limitations, we found that only one strain adapted to colistin selective pressure—the strain that expressed functional MCR-3. This is an important finding because it suggests that subinhibitory levels of antimicrobials might be more effective in facilitating the acquisition of resistance in some strains, for example, those carrying existing genes that confer reduced susceptibility. However, it is important to note that the mutations necessary for acquired colistin resistance come at a significant fitness cost to the bacterium. While the mutations detected in our populations increased the competitive performance of strains under colistin selection, these strains had lower fitness in other contexts, that is, when grown in standard media or in the presence of human serum. The differences in context-dependent fitness could explain why the mutated alleles that primarily drove phenotypic outcomes in the evolved *mcr-3* strains, that is, *lpxC* and *hns*, were only rarely mutated in natural *E. coli* isolates. Our results also revealed *hns1* mutation as the only predictor of MCR-3 protein level, suggesting that H-NS directly or indirectly controls MCR-3 expression. This could explain the lack of fitness cost observed in natural *mcr*-carrying *E. coli* due to H-NS-mediated silencing of MCR-3 expression. This, however, would need to be experimentally validated for *mcr-3* as well as for other *mcr* family genes.

## MATERIALS AND METHODS

### Bacterial strains, plasmids, and growth conditions

All bacteria and plasmids used in this study can be found in Tables S3 and 4, respectively. *E. coli* strains were grown at 37°C, 200 rpm in Difco LB Lennox Broth (LB; Becton, Dickinson and Company [BD], Franklin Lakes, NJ) or in BHI Broth (BD) as indicated. For *E. coli* strains carrying the pTnS2, pUC18R6K, or pUC19 plasmids, 100 μg/mL of ampicillin (Amp) was added to the culture medium. For *E. coli* strains carrying the pCP20 plasmid, 30 μg/mL of chloramphenicol was added to the culture medium.

### Construction of single-copy *mcr-3* and *mcr-9* in *E. coli* MG1655

To make single copies of *mcr-3* and *mcr-9* with their native promoters and a fused MYC-tag, each gene, including 127 and 167 bp upstream of the start codon, was PCR amplified with the AS2635_8 and _9 and AS2635_10 and _11 primers (see Table S5) from genomic DNA of *Salmonella enterica* isolates FSL R9-3269 and FSL R9-3274, respectively. Primers AS2635_9 and _11 included MYC-tags. PCRs were performed with Q5 high-fidelity DNA polymerase (New England Biolabs [NEB], Ipswich, MA). PCR products and the pUC18R6KT-mini-Tn7T plasmids were digested with EcoRI-HF and KpnI-HF or EcoRI-HF and SacI-HF (NEB), ligated with DNA ligase (NEB), transformed into *E. coli* Pir1 strains via heat shock, and selected by plating on Amp-supplemented LB agar plates, all according to the manufacturer’s recommendations. The correct insertion of genes into the *attTn7* locus was confirmed via Sanger sequencing with the AS2635_12 and _13 primers by the Biotechnology Resource Center (BRC, Cornell University, Ithaca). The correctly assembled plasmids pUC18R6KT-mini-Tn7T (empty control), pUC18R6KT-mcr-3-Myc, or pUC18R6KT-mcr-9-Myc were co-transformed with helper plasmid pTnS2 at equal volumes into *E. coli* K-12 MG1655 strains via heat shock. Successful Tn7 integration was selected for by plating on LB agar plates with 50 μg/mL kanamycin (Kan). Correct insertion of the *mcr-3* and *mcr-9* transposon was confirmed first via Sanger sequencing with the AS2635_14 and _23 primers flanking the *attTn7* insertion site by the BRC, followed by DNA isolation and whole-genome sequencing (WGS) of the final mutants (S14-0047 and -0049, respectively), as described below.

### *In vitro* experimental evolution

Single colonies of *E. coli* K-12 MG1655 *attTn7::mini-Tn7-Km (E. coli* empty*)*, *-mcr-3-Km*, (*mcr-3),* or *-mcr-9-Km* (*mcr-9)* were inoculated in BHI broth (BD Difco) and grown for 15 to 18 h overnight. To start the *in vitro* evolutionary experiment, 1:1,000 of each overnight culture was inoculated into (i) six culture tubes containing BHI broth with different concentrations of colistin ranging from 0.25–8 μg/mL in twofold steps and (ii) a seventh culture tube containing BHI broth without colistin (positive control). After 24 h of incubation, the culture tube with the highest colistin concentration that showed growth was selected for further propagation. This culture tube was used to (i) prepare 15% (vol/vol) glycerol stocks for downstream DNA isolation and MIC determination, (ii) inoculate new culture tubes containing BHI broth with different colistin concentrations at 1:1,000, and (iii) the remaining broth was spun down at 6,000 rpm for 10 min (Sorvall X4RF PRO-MD centrifuge), and saved at −80°C for Western blot analysis of protein expression. The culture tubes used for propagation always included one tube containing BHI without colistin as a growth control and six tubes containing BHI with different colistin concentrations in twofold increments (see Fig. 2). Colistin concentrations were chosen based on the concentrations that showed growth on the previous day and were increased or decreased as needed. The procedure was repeated for a total of 7 days for the *E. coli* MG1655 empty control propagation. For the *E. coli* MG1655 *mcr-3* propagation, the day 4 culture was used to inoculate two new culture tubes, which were then passaged separately in colistin (as described above) for another 3 days. Afterward, each propagation was passaged every 24 h in the absence of colistin by passaging 1:1,000 of the previous day’s population into a culture tube containing BHI for another 7 days. For the propagation in the absence of colistin, only the populations from the last day were stored as glycerol stocks and used to collect pellets for Western blot detection of protein expression. The *E. coli* MG1655 *mcr-9* propagation was repeated for 14 days in the presence of colistin as described above.

### DNA extraction, sequencing, and bioinformatic analysis

For parent and evolved *mcr-3* strains, single colonies were inoculated into LB broth with appropriate antibiotics; for populations from the *in vitro* evolutionary experiment, 25 μL of the saved glycerol stocks were inoculated in 5 mL of BHI broth supplemented with 50 μg/mL of Kan. All strains and populations were grown for 15–18 h overnight. DNA from cultures was extracted using a QiAamp DNA Mini Kit (Qiagen, Germantown, MD) according to the manufacturer’s instructions. Illumina NextSeq 500 platform (Illumina, Inc., San Diego, CA), with 2 × 150 bp paired-end reads was used to sequence genomic DNA. All populations and isolates that were sequenced can be found in Table S6; all sequencing data are uploaded under BioProject ID PRJNA1336184. Raw sequencing data were processed using the software Trimmomatic v.0.39 (52) to trim adapters and remove low-quality bases, followed by quality assessment using FastQC v.0.11.8 (53). SPAdes v.3.15.2 (54) in careful mode was used to assemble reads of strains S14-0047 (*E. coli* MG1655 *mcr-3*) and S14-0049 (*E. coli* MG1655 *mcr-9*); quality of assemblies was checked using QUAST v.5.0.2 (55), and Geneious Prime v.2024.0.5 was used to confirm successful insertion of *attTn7::mcr-3* and *::mcr-9*.

Sequencing results of the isolated DNA were used to check for potential contamination of our populations during the *in vitro* evolutionary experiment. Additionally, nonsynonymous mutations and small deletions were identified in (i) populations from the *in vitro* evolutionary experiment as well as (ii) parent strains and evolved *mcr-3* strains by comparing the trimmed raw reads to the *E. coli* MG1655 reference genome (NCBI accession number NC_000913) using breseq v.0.39.0 (22). The polymorphism mode was used for populations, and the consensus mode was used for parent and evolved strains. All mutations that were detected in (i) both the parent strain (e.g., *mcr-9* parent strain) and the resulting populations (e.g., *mcr-9* populations), and (ii) both the *mcr-3* parent strains and the evolved *mcr-3* strains were removed from the results.

### Isolation of evolved *mcr-3* strains

To analyze how mutations identified in the breseq analysis impact bacterial phenotypes, evolved *mcr-3* strains were isolated from the different populations that evolved during the *in vitro* evolutionary experiment (see Table 1). A total of six strains were isolated, which represented all possible combinations of individual mutations that occurred during propagation A or B. Glycerol stocks containing the different populations were streaked onto LB plates supplemented with 50 μg/mL of Kan and were incubated overnight for 15–18 h. Using sterile toothpicks, individual colonies were first picked onto LB plates supplemented with 50 μg/mL of Kan for storage, followed by picking colonies into PCR tubes containing dH_2_O and heating the PCR tubes at 95°C for 10 min. Picked colonies were tested with *de novo*-created *hns*, *lpxC*, *pmrB*, *waaQ*, *sbmA*, *degS*, and *dnaJ* primers (AS2635_24 to _37, Table S5), each amplifying a 230 bp–400 bp long PCR product around the mutated site. Because the *pmrB1* and *waaY1* mutations occurred at the same time, we did not screen for the *waaY1* mutation. All PCR reactions were run using the GoTaq Green Master Mix (Promega; Madison, WI) under the following conditions: initial denaturation for 2 min at 95°C, 30 cycles of denaturation for 30 s at 95°C, annealing for 30 s at 53°C, and extension for 1 min at 72°C, followed by a final extension for 5 min at 72°C. Remaining primers and deoxynucleotide triphosphates were digested by adding shrimp alkaline phosphatase (Thermo Fisher) and exonuclease I (Thermo Fisher) and incubating the mixture for 45 min at 37°C, followed by inactivation of the enzymes for 15 min at 80°C. PCR products were sequenced by Sanger sequencing at the BRC. Mutations were confirmed by aligning each sequenced fragment to the *E. coli* MG1655 reference sequence in Geneious Prime v.2024.0.5.

The genotypes of all evolved *mcr-3* strains were confirmed via WGS (see above); raw reads of all evolved *mcr-3* strains can be found under BioProject ID PRJNA1336184, individual BioSample numbers can be found in Table S6.

### Colistin susceptibility testing

Colistin susceptibility was tested using broth microdilution based on CLSI guidance (56), except for using PlateONE polypropylene 96-well plates (USA Scientific; Ocala, FL). Overnight cultures of parent and evolved strains were prepared by inoculating single colonies in LB broth. Overnight cultures of populations were prepared by inoculating 25 μL of the saved glycerol stocks in 5 mL of LB broth. Overnight cultures were grown for 15–18 h, subsequently diluted 1:200 in BBL Mueller-Hinton II cation-adjusted broth (MHII, BD), and incubated until cultures reached an OD_600_ of 0.2–0.4. Populations and strains were inoculated at final concentrations of 5 × 10^5^ CFU/mL into MHII broth containing colistin (0.125 × 128 μg/mL, twofold dilutions) and grown statically at 35°C for 16×20 h. All populations, parent strains, and evolved *mcr-3* strains were tested in biological triplicates.

### Lipid A analysis

To determine lipid A structures for the evolved *mcr-3* strains, all evolved *mcr-3* strains, the *mcr-3* parent strain, and the empty control strain were grown overnight for 12–18 h in LB broth, followed by 1:200 back dilution in BHI broth and incubation for 6 h. Cells were collected in 5 mL aliquots by centrifugation at 6,000 rpm for 5 min (Sorvall X4RF PRO-MD centrifuge). The supernatants were removed, and pellets were stored at −80°C prior to lipid A analysis. Lipid A structural analysis was performed as previously described (57). Bacterial pellets were spotted onto a stainless steel 96-well MALDI target plate, followed by the addition of 1 µL of FLAT extraction solution (0.2 M anhydrous citric acid, 0.1 M trisodium citrate dihydrate). The FLAT target plate was incubated at 100°C in a humidified heat block for 30 min. Bacterial spots were washed with endotoxin-free water (Gibco, Grand Island, NY, USA) and air-dried, followed by the addition of norharmane matrix (10 mg/mL in 1:2 MeOH:CHCl_3_ – Sigma-Aldrich, St. Louis, MO, USA). Mass spectra were collected in negative-ion mode using a Bruker Microflex LRF within the 1,000–2,400 m/z range, 300 shots. Agilent ESI Tune Mix was used as an external calibrant. MALDI-TOF MS data were processed and analyzed with flexAnalysis software (version 3.4).

### Western blot analysis

Western blot analyses were performed to confirm MCR-MYC expression, as previously described (58, 59). Samples for populations were collected as described in the “*In vitro* evolutionary experiment” section. To collect samples for evolved *mcr-3* strains, strains were grown for 15–18 h overnight in LB broth, back diluted 1:200 into BHI broth, incubated for 6 h, and collected via centrifugation at 6,000 rpm for 10 min (Sorvall X4RF PRO-MD centrifuge) and stored at −80°C. The collected pellets were resuspended in SDS-PAGE lysis buffer and denatured by heating at 95°C for 10 min, followed by sonication. Lysed cells were resolved on 4% to 20% Mini-Protean stain-free TGX precast protein SDS-PAGE gels (Bio-Rad Laboratories; Hercules, CA). Total protein bands were visualized after UV light exposure for 1.5 min using the Bio-Rad ChemiDoc MP Imaging system. The proteins were then transferred to polyvinylidene difluoride (PVDF) membranes, followed by blocking of membranes with TTBS (TBS buffer [50 mM Tris-Cl, 150 mM NaCl, pH 7.5] with 0.1% [vol/vol] Tween 20) containing 5% Blotting-Grade Blocker (Bio-Rad Laboratories) for 30 min. Membranes were then incubated with a rabbit anti-MYC primary antibody (Sigma-Aldrich; at 1:500 dilution) diluted in TTBS with 0.5% (wt/vol) Blotting-Grade Blocker, at room temperature overnight with gentle shaking. The membranes were washed with TTBS, followed by incubation with goat anti-rabbit horseradish peroxidase secondary antibody (Thermo Fisher Scientific, at 1:3,000 dilution) for 2 h at room temperature with gentle shaking. The membranes were washed with TTBS and TBS before development with the Clarity Western ECL Substrate (Bio-Rad Laboratories) and subsequent visualization using the Bio-Rad ChemiDoc MP Imaging system. MCR-3 protein levels were semi-quantitatively analyzed by estimating the area under the curve of MCR-3-MYC bands visible on the PVDF membrane and all bands visible on the SDS-PAGE gel using densitometric analysis of bands with the Bio-Rad Image Lab 6.1 software as previously described (58). Data were analyzed using a one-way ANOVA with *post hoc* Dunnett’s test.

### Bacterial growth assessment

Bacterial growth of parent strains and evolved *mcr-3* strains was assessed as previously described (59). Overnight cultures were prepared by inoculating single colonies in LB broth and grown for 15-18 h. Overnight cultures of parent strains were diluted 1:200 in MHII and grown for 24 h. After 0, 2, 4, 12, and 24 h of incubation, 200 μL of each culture was removed, and 10-fold serial dilutions were performed in phosphate-buffered saline (PBS). Overnight cultures of evolved *mcr-3* strains were diluted 1:200 in BHI broth and BHI broth supplemented with 5% (vol/vol) human serum (Sigma-Aldrich) and grown for 24 h. After 0, 4, 6, and 24 h of incubation, 200 μL of each culture was removed, and 10-fold serial dilutions were performed in PBS. For each dilution (10^0^–10^7^), 10 μL volumes were spotted onto LB broth agar plates (BD) (prepared with 15 g/L Bacto Agar [BD]) and incubated overnight. Colonies were counted to determine CFU/mL, and data were plotted on a log scale. All strains and conditions were assessed in biological triplicates.

### Competition experiments to assess context-dependent fitness and competitive performance

The *E. coli* MG1655 empty strain was used to calculate the relative fitness (assays in BHI and BHI supplemented with human serum) and relative competitive performance (assay under colistin selection) of the *mcr-*3 parent strain and each evolved *mcr-3* strain. *E. coli* MG1655 *mcr-3* parent and evolved strains, in addition to the *E. coli* MG1655 empty strain, competed against *E. coli* MG1655 pUC19, which produces blue pigment when grown on media containing 5-bromo-4-chloro-3-indoyl β-D-galactopyranoside (X-Gal) (Gold Biotechnology; Olivette, MS). Overnight cultures were prepared in LB broth as previously described, grown for 12–18 h, and diluted 1:10 in LB broth. The optical density (OD) of diluted overnight cultures was measured at 600 nm to estimate the concentrations of the cultures. *E. coli* MG1655 empty, *mcr-3*, or evolved *mcr-3* strains were added to BHI broth, BHI broth containing 2 μg/mL of colistin, or BHI broth containing 5% (vol/vol) human serum (Sigma-Aldrich) at a 1:1 ratio with competitor strain *E. coli* MG1655 pUC19 at a final concentration of 2.5 × 10^5^ CFU/mL per strain. We plated 10-fold serial dilutions of all strains before the competition to determine initial bacterial concentrations. Competition experiments were carried out for 6 h at 37°C and 200 rpm, followed by plating 10-fold serial dilutions made in PBS on LB agar plates supplemented with 200 μg/mL of X-Gal. Plates were incubated overnight, and white and blue colonies were counted in the countable range (20–200 colonies/plate). Competition assays were performed in biological triplicates. We used the following formula to calculate fitness/competitive performance: fitness/competitive performance = [final concentration (white) / initial concentration (white)] / [final concentration (blue) / initial concentration (blue)]. Fitness/competitive performance values were adjusted based on the fitness/competitive performance value of the *E. coli* MG1655 empty strain. For the competition assays in the presence of colistin, we added one colony to each count because we could not recover cells for some of the assays. Statistics were performed on log_10_-transformed fitness values for the competition in the presence of human serum and log_10_-transformed competitive performance values for the competition under colistin selection, so data were normally distributed; mean relative fitness/competitive performance values of evolved *mcr-3* strains were compared to the *mcr-3* parent strain by a one-way ANOVA with post-hoc Dunnett’s test.

### Correlation analysis and linear regression models

To analyze whether the phenotypic outcomes of the *mcr-3* parent and evolved *mcr-3* strains were correlated, Pearson correlation coefficients were calculated for the following data: MCR-3 protein level, bacterial growth (BHI) data after 4 h, log_10_-transformed bacterial growth (human serum) after 4 h, fitness (BHI), log_10_-transformed competitive performance (colistin), log_10_-transformed fitness (human serum). Log_10_-transformed data were utilized when untransformed data were not normally distributed. Pearson’s correlation coefficients were visualized in the correlation matrix if they were determined to be significant (*P* < 0.05) using the corrplot package (31).

To determine how nonsynonymous mutations impacted various phenotypes, linear regression models were run. The mutations *hns1*, *lpxC1*, *waaQ1*, *waaY1_pmrB1*, *dnaJ1*, and *sbmA1_degS1* were encoded as binary variables, with 0 meaning that the mutation was not present and 1 meaning that the mutation was present. The variables *waaY1* and *pmrB1*, as well as *sbmA1* and *degS1,* occurred simultaneously, so each of the two mutations was represented as one variable. Additionally, *hns2* was encoded as *hns1* = 0, as the *hns2* mutation likely reverts the effects of the *hns1* mutation. Using the established six predictors, linear regression models were run for each outcome of (i) MCR-3 protein level, (ii) bacterial growth in BHI and in the presence of human serum, and (iii) fitness in BHI, in the presence of human serum, and competitive performance under colistin selection. The fit of the linear regression models was optimized using the step function in both directions.

### Conservation analysis of *hns* and *lpxC* in diverse *E. coli* strains

To test the general conservation of *hns* and *lpxC* in diverse *E. coli* strains, a file of all available *E. coli* genomes (*n* = 439,279) was downloaded from NCBI Pathogen Detection (22 November 2024). All entries without SNP clusters and assembly numbers were removed. Out of the resulting 38,201 entries, 2,000 random SNP clusters were selected, from which one representative isolate was selected. The genome assemblies of a total of 1,999 representative isolates were downloaded on November 22nd, 2024, as the assembly of one of the 2,000 chosen isolates was not available (Sup. Table 2). To ensure that the randomly selected isolates represented a large diversity, sequence types were assigned for each isolate using mlst v.2.23.0 (60). To compare SNPs and nonsynonymous mutations in *hns* and *lpxC*, the gene sequences for the *hns* and *lpxC* genes were isolated from each strain based on the *E. coli* K-12 MG1655 reference sequences (NCBI genome accession number NC_000913, locus tags: b0096 [*lpxC*] and b1237 [*hns*]) using NCBI nucleotide BLAST v.2.16.0 (61). All *lpxC* and *hns* sequences were aligned in Geneious Prime v.2024.0.5 to determine the number of SNPs (Sup. Table 2). All *lpxC* and *hns* gene sequences were also translated into amino acid sequences and aligned in Geneious Prime v.2024.0.5 to determine the number of nonsynonymous mutations (Sup. Table 2). To identify *mcr-*carrying strains in our 1,999 randomly selected strains, metadata were downloaded from NCBI Pathogen Detection (downloaded 4 December 2024). We considered an isolate to carry an *mcr* variant if an *mcr* variant was listed as “complete” in the “AMR genotype” column.

### Statistical analysis

All statistical analyses were performed using R Statistical Software v4.1.2 (R Foundation for Statistical Computing, Vienna, Austria).

## SUPPLEMENTAL MATERIAL

Supplemental figures aac.00457-26-s0001.pdf Fig. S1 to S3.

Supplemental tables aac.00457-26-s0002.xlsx Tables S1 to S6.

ASM does not own the copyrights to Supplemental Material that may be linked to, or accessed through, an article. The authors have granted ASM a non-exclusive, world-wide license to publish the Supplemental Material files. Please contact the corresponding author directly for reuse.
