# Supplementary material for: Acquired mutations in transcriptional regulator hns and lipopolysaccharide biosynthesis and modification genes impact colistin resistance and fitness of mcr-carrying E. coli
Source: Antimicrob Agents Chemother. 2026 Jun 18;70(8):e00457-26. doi: 10.1128/aac.00457-26 (PMC13436332; doi:10.1128/aac.00457-26)
Supplement: Supplementary file 1 [file aac.00457-26-s0001.pdf]

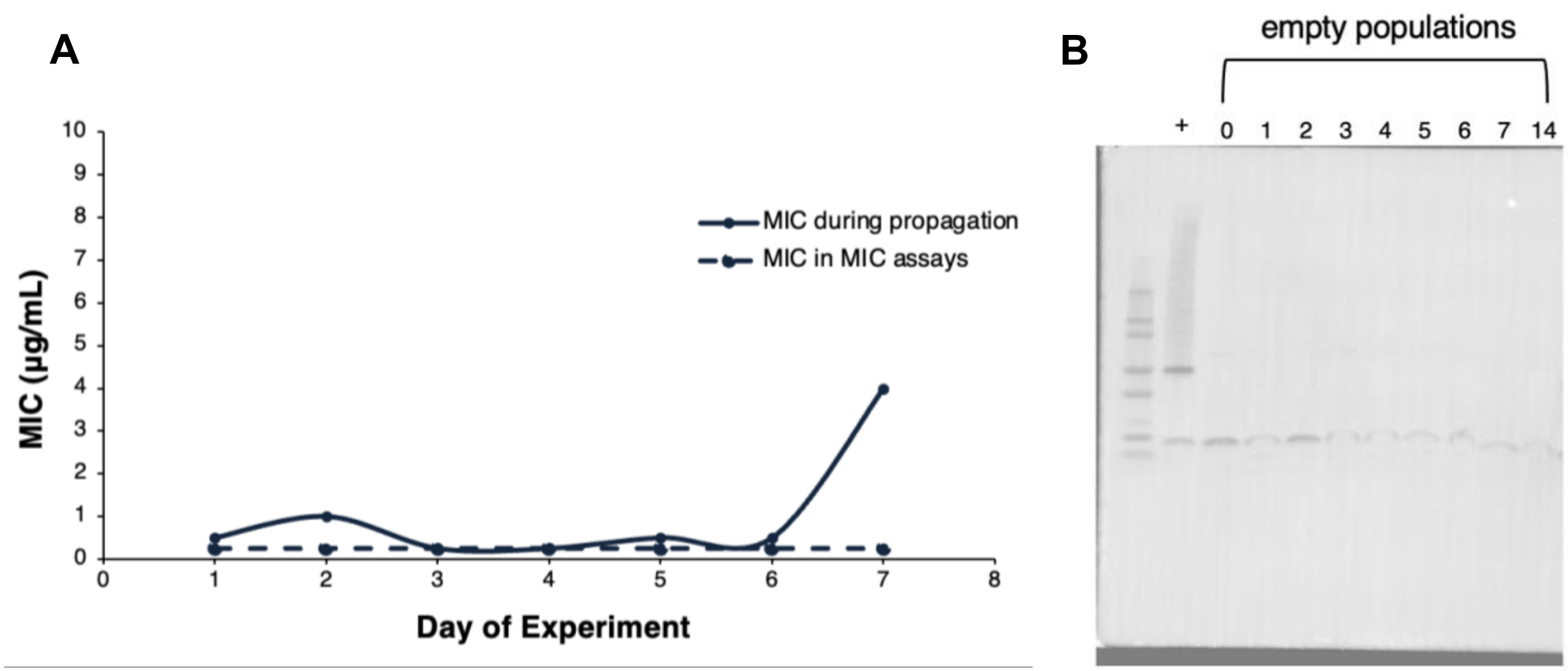

Sup. Figure 1. Colistin MICs and protein expression of *E. coli* empty populations throughout *in vitro* evolutionary experiment.

(**A**) Colistin MICs of *E. coli* MG1655 empty populations throughout the propagation experiment (n=1; i.e., the lowest concentration where no growth was observed) and assessed in MIC assays later (n=3, data points represent means). (**B**) Western blots of *E. coli* MG1655 empty populations collected at the end of every day of the propagation experiment. Each day is denoted by a number, day 0 represents the overnight culture from the input/parental strains. The "+ ctrl" represents *E. coli* MG1655 *mcr-3-myc* fusion strains known to express MCR-3-MYC from initial experiments (data not shown).

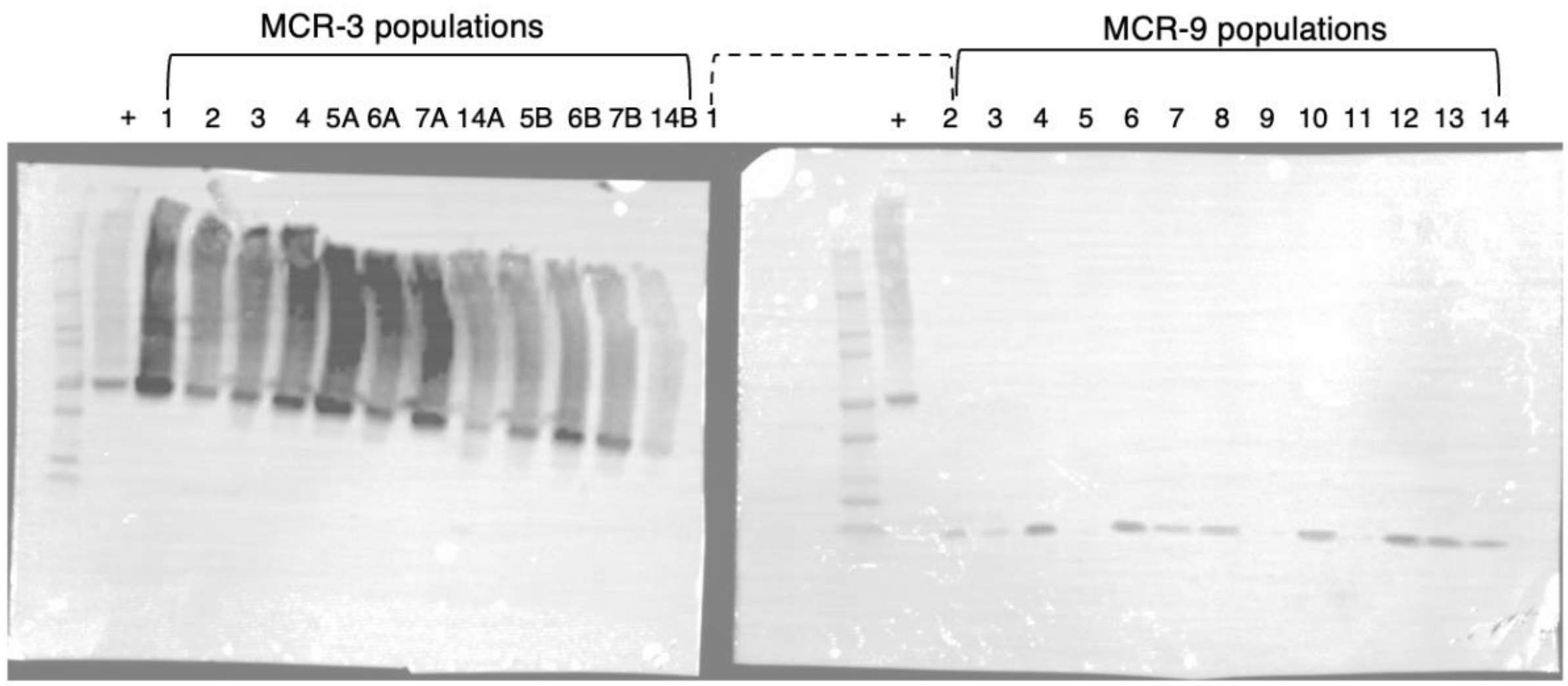

Sup. Figure 2. Protein expression of *E. coli mcr-3* and *mcr-9* populations throughout *in vitro* evolutionary experiment.
Western blots of *E. coli* MG1655 *mcr-3* and *mcr-9* populations collected at the end of every day of the propagation experiment. Each day is denoted by a number, day 0 represents the overnight culture from the input/parental strains. For *E. coli* MG1655 *mcr-3* populations, “A” and “B” denote the populations of when the population was split and propagated separately. The “+ ctrl” represents *E. coli* MG1655 *mcr-3-myc* fusion strains known to express MCR-3-MYC from initial experiments (data not shown).

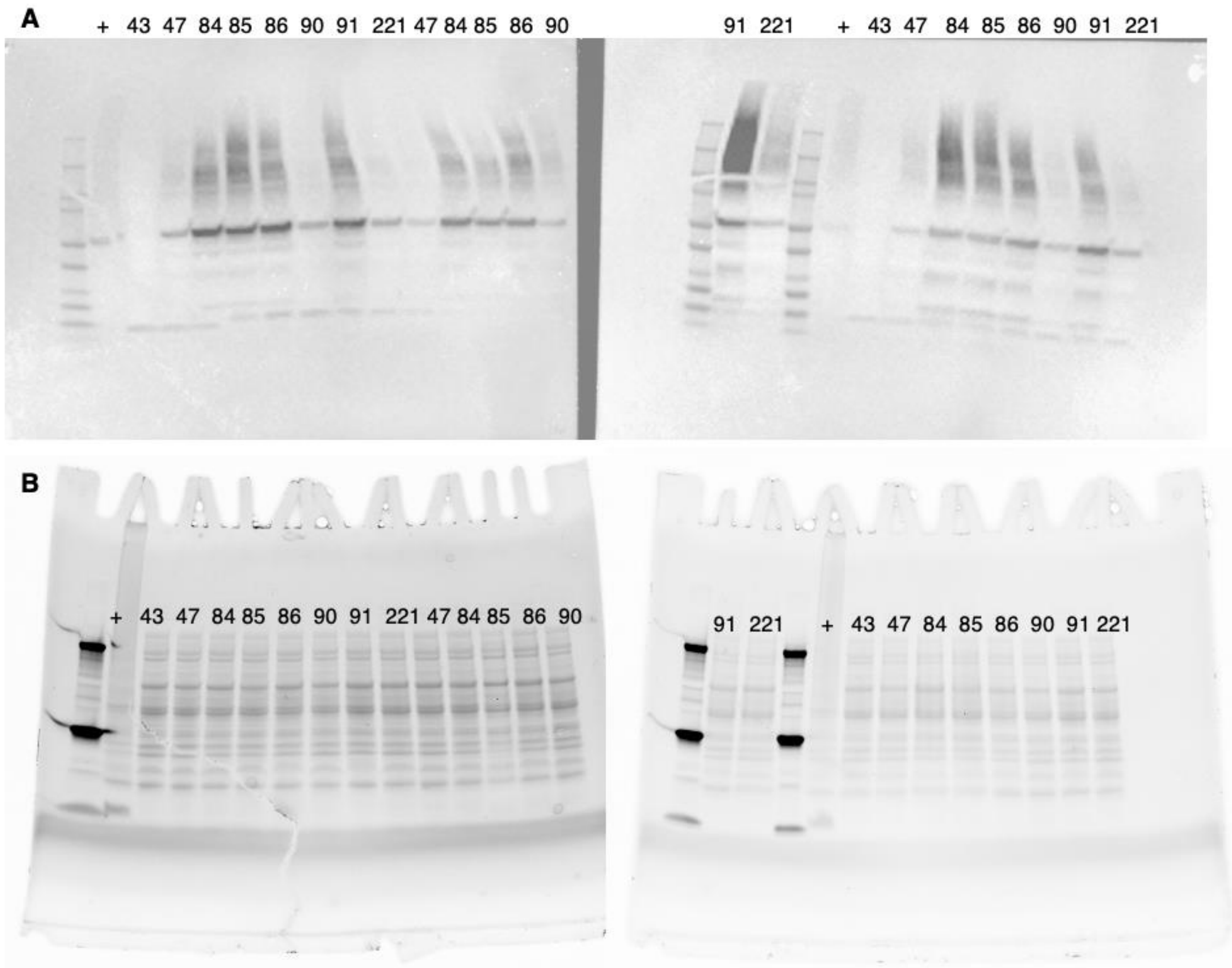

Sup. Figure 3. Protein expression of *E. coli mcr-3* individual strains.
(A) Western blots and (B) SDS page gels of *E. coli* MG1655 *mcr-3* individual strains used to assess total MCR-3 protein expression semi-quantitatively. The "+ ctrl" represents *E. coli* MG1655 *mcr-3-MYC* fusion strains known to express MCR-3-MYC from initial experiments (data not shown).
